# An Approved Landing Site (ALS) improves the logistics of interhospital transfer of critically ill patients by helicopter

**DOI:** 10.1186/s13049-021-00894-2

**Published:** 2021-08-03

**Authors:** Geert-Jan van Geffen, Ed J. Spoelder, Amanda Tijben, Cornelis Slagt

**Affiliations:** 1 Department of Anesthesiology, Pain and Palliative Medicine and Helicopter Mobile Medical Team Lifeliner 3 and 5, Radboud University Medical Centre, P.O. Box 9101, Geert Grooteplein Zuid 10, 6500 HB Nijmegen, the Netherlands; 2 ANWB-Medical Air Assistance , Emoeweg 16, 8218 PC Lelystad, the Netherlands

## Abstract

The COVID-19 pandemic limited hospital resources and necessitated interhospital transport of ICU-patients in order to provide critical care to all patients in the Netherlands. However, not all hospitals have an approved landing site. The ICU-transport operation was executed under HEMS-license and landing on non-aerodrome terrain was permitted. This allowed the search for an ad-hoc landing site in the direct vicinity of the ICU. The following characteristics were judged: slope, obstacles, size, soil conditions and the presence of foreign objects.

Before the start of this transport operation, in two days, all hospitals in the Netherlands were visited and presumed landing sites explored, described, photographed and recorded in the electronic flight bag. At 71 (87,6 %) of the hospitals it was possible to install a temporary approved landing site in the direct vicinity of the ICU. 110 landings were made on these landing sites and 114 landings on approved heliports. Only 11 patients required secondary transport to or from the helicopter landings site. This occurred only in two patients from a heliport to a receiving hospital.

The construction of pre-explored approved landing sites in the vicinity of hospitals allows safe transportation of patients by helicopter to hospitals without a heliport.

**Supplementary Information:**

The online version contains supplementary material available at 10.1186/s13049-021-00894-2.

## Introduction

In the Netherlands four physician-staffed emergency helicopter mobile medical teams provide prehospital emergency care for critically ill or severely injured patients. The helicopter is mainly used for transporting the medical team, consisting of a physician (anesthesiologist or trauma-surgeon) and flight-nurse, to the scene. Patients are transported by helicopter if this is more time-efficient compared with ground transport by ambulance.

The COVID-19 pandemic limited intensive care resources and necessitated interhospital transport of ICU-patients in order to provide critical care to all patients. Especially for long distance transfers (> 100 km) helicopter transport (Lifeliner 5) was added to the regular means of ground transportation by Mobile Intensive Care Units (MICUs). Transporting patients by helicopter is fast, efficient, comfortable and safe with regard to the crewmembers [1]. The decision to transport patients by helicopter or MICU was made by the National Coordination Centre for Patient Evacuation (LCPS).

In this paper we describe the logistics and planning of inter-hospital helicopter transport of COVID-19 infected ventilated intensive care patients in the Netherlands with regard to helicopter landing sites.

## Landing sites and certification

In the Netherlands the emergency medical helicopter provider, ANWB Medical Air Assistance (ANWB MAA), possesses a Helicopter Emergency Medical Services (HEMS)-license for providing medical care as is required according to the European Regulations on “Air Operations” (9665/2021). This license gives exemptions and dispensations. For these air operations apply other weather minimums than regular commercial flights. The helicopters may gain entrance to regulatory special use airspace as prohibited and restricted areas. Regarding the urgent character of a HEMS-flight, dispensation for providing a flight plan is granted and the helicopters mostly gain directly permission to leave for a mission. The HEMS-helicopters have a permanent waiver for landing on non-aerodrome terrain.

In the Netherlands most hospitals do not have an officially approved helicopter landing site according the International Civil Aviation Organization, (ICAO) Annex 14 standard [2]. This annex contains standards and recommended practices (SARPs) for airport design and operations such as physical characteristics, obstacle limitations and facilities and technical services normally provided at an aerodrome. The associated costs for constructing and maintaining an approved helipad is an important reason for not constructing an aerodrome in the vicinity of the hospital. Moreover the urge to have a pad depends on the characteristics of the provided care. While regional and small hospitals do not have approved landing sites (ALS), all Dutch trauma centers possess an aerodrome.

The COVID-19 pandemic necessitated ICU-patient transfers from all hospitals regardless the principal care they provide. The ICU-transport operation was executed under HEMS-license and landing on non-aerodrome terrain was permitted. This allowed the search for an ad-hoc landing site in the direct vicinity of the ICU.

In order to improve flight safety, assumed landings sites in the direct vicinity of the ICUs were explored by ANWB-MAA pilots and security officers of the hospitals. Characteristics were described, photographed and together with a geographical map recorded in the electronic flight bag (EFB). All hospitals were visited and approved landing sites were described.

## Helicopter landing sites requirements and flight safety

During landing of the helicopter a presumed landing site is judged by the pilot and Helicopter Crew Member (HCM) nurse whether the location is fit for landing regardless whether the landing site is officially approved or not. The following characteristics are judged; slope, obstacles, size, soil conditions and foreign objects.

The slope of the surface should be within pre-determined limits and is dependent on the type of helicopter. It should prevent mechanical overloading or a rolling over of the aircraft. For the H145 (The ICU-transport helicopter) this is 10° forward, 8° backward, 8° to the left and 12° to the right.

**Fig. 1:**
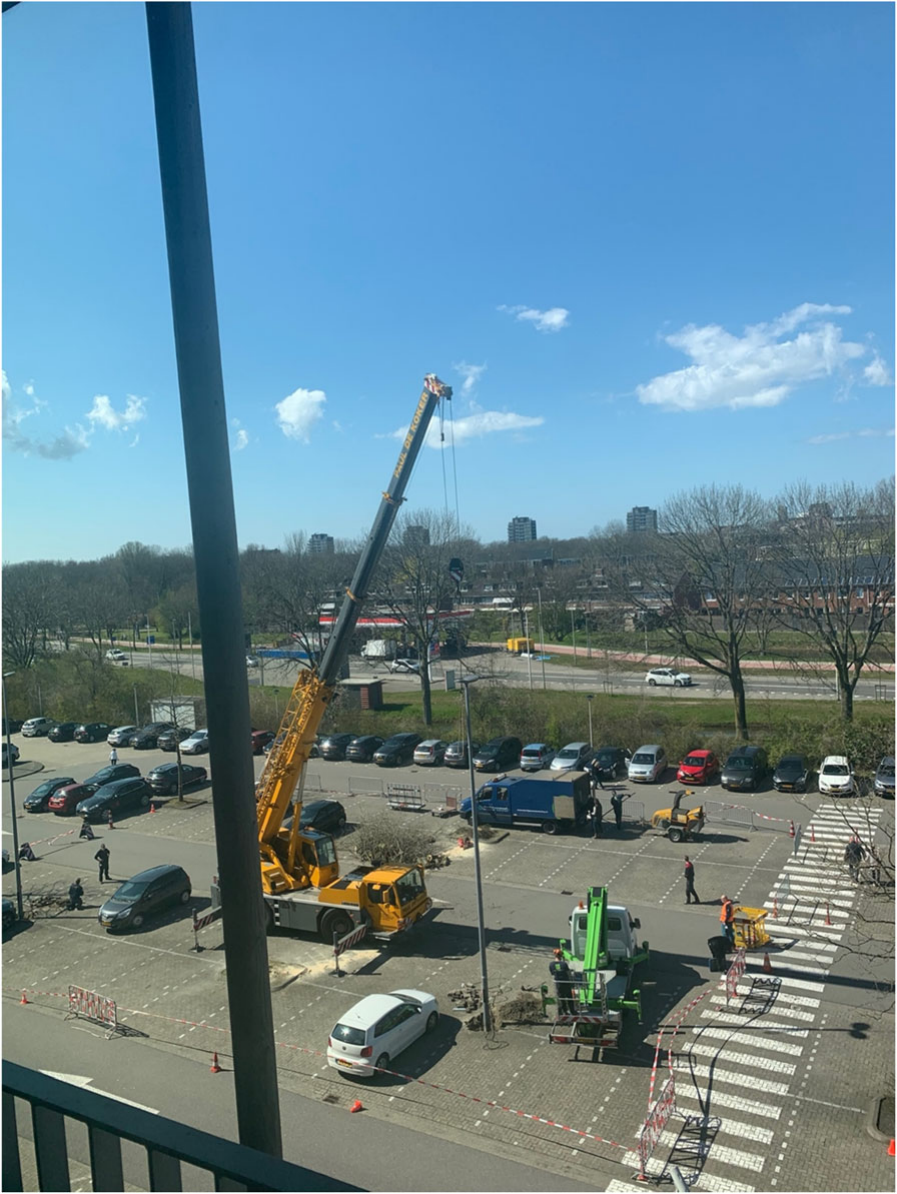
Construction of an ALS at the parking lot of Alrijne Hospital, Leiderdorp, the Netherlands

A landing site surrounded by high trees, lamppost or other high objects should be avoided because of safety reasons during take-off and landing. Figure 1 shows the designing of an ALS which allowed landing on a parking lot in close vicinity of the ICU.

The size of the landing site depends on the type of helicopter and is dependent on the total length of the aircraft. For the H145 this is 28 m x 28 m. During night time this is doubled, 28 m x 56 m. due to the limited view. The soil conditions are not only judged because of avoidance of sinking of the helicopter, but also because of the air displacement (downwash), dust, sand, grit etc. can be blow up into the air. Blow up of drifting snow (white-out) or drifting sand can blur the view of the pilot and cause danger to the crew and injure by-standers. Loose objects may cause damage in the environment or helicopter due to air displacement. Preferably landing sites should not be marked by freestanding fences, pawns, or marking ribbons, because they might be blown away and be a risk for the helicopter and surroundings. Finally, the site is inspected for humans and animals. The loud sound of the engine may frighten animals. Runaway animals may injure themselves or others and cause damage.

**Fig. 2:**
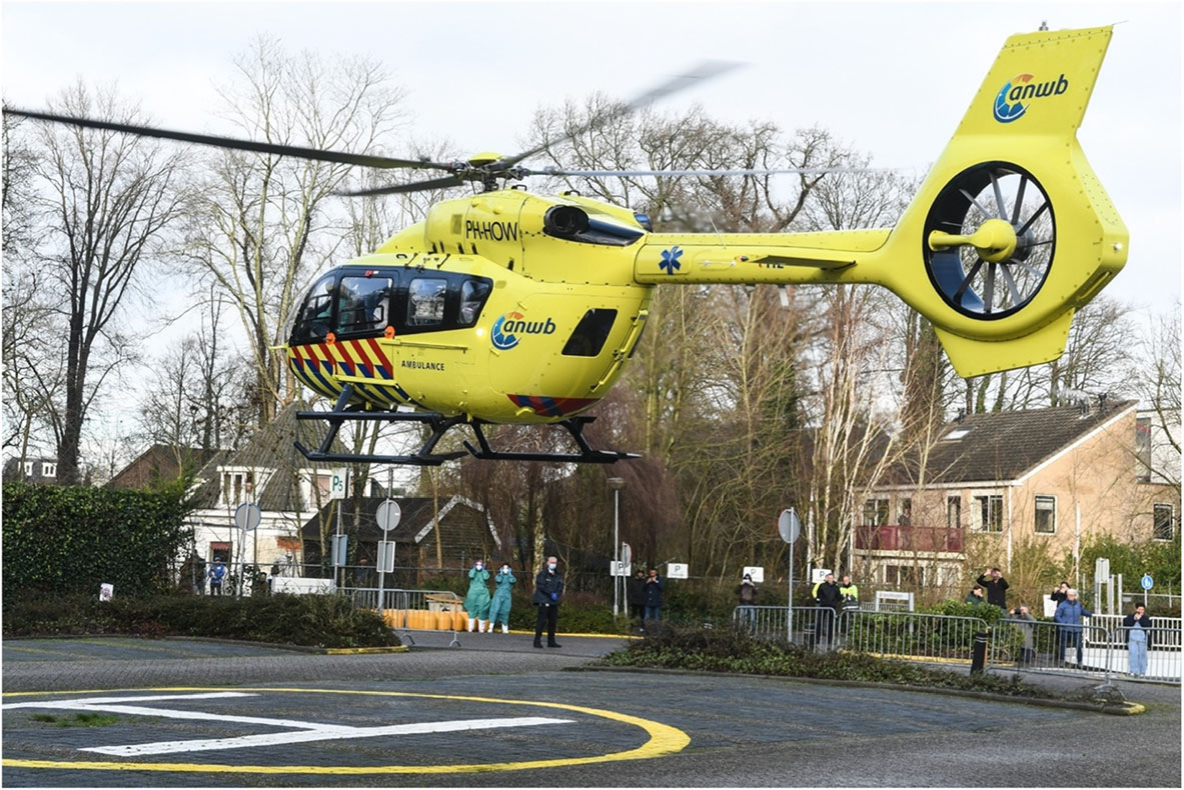
Landing at an ALS

During night, the view on the landing site is limited. Visual aids in order to improve night view such as night vision goggles are hindered by strong ambient lights. This is among other safety precautions, the reason not to make HEMS-landings in contiguous built-up areas during night-time. However, a HEMS-landing on a pre-explored and approved landing site in the direct vicinity of a hospital in a city is allowed (Fig. 2).

Strictly applied, no additional security or fire brigade is necessary on an ALS-location, although it is advised to inform them about the planned flight. They might inspect the landing site and judge whether it still fulfills the requirements for a save landing zone. Obstacles and objects can be removed and during landing they can guide bystanders and provide additional safety.

## Approved landing sites requirements and patient safety

For ICU-patient transportation additional requirements apply for a landing site. The distance between intensive care (IC) and emergency department should be as short as possible. The road from hospital to ALS should be paved and allow easy cornering and steering of the patient transport stretcher with medical equipment which may weigh up to 250 kg. Preferably the road could be shielded because of patient privacy.

## Avoidance of secondary transport by ambulance

During the second wave of the COVID-19 pandemic the helicopter transport operation was restarted after an interruption of several months and it was noted that some hospitals changed the hospital area and reinstalled paying parking places on presumed landing sites. This necessitated additional transportation from the ICU to the helicopter landing site. Not only the total transport time increases, but also the risk on adverse effects [3].

Aviation certified transport trolleys are not compatible with ground ambulances and require additional patient transfers or additional organization and logistics.

## Experiences

An ALS was designated at 71 ( 87,6 % ) of the general hospitals in the Netherlands (Fig. 2). Dense buildings, obstacles, or lack of cooperation to prepare parking places for helicopter landing prevented the designation of an ALS in the direct vicinity of 10 hospitals.

Between march 24 2020 and march 24 2021, in the Netherlands, 112 interhospital helicopter transfers of COVID-19 ICU-patients were made (Table 1). During this one year period 110 (49,1 %) landings were made at temporary landing locations, (See video for helicopter landing at a temporary ALS.) Secondary transport by ambulance from hospital to a landing site or vice versa was performed in 11 (4.9 %) patients.


Additional file 1

**Table 1:** Landing site characteristics of interhospital and secondary transport in the Netherlands

Landings at referral hospital (n = 112)				Landings at receiving hospital (*n* = 112)			
**ALS**		**HLS**		**ALS**		**HLS**	
N	%	N	%	N	%	N	%
84	75.0	28	25	26	23.2	86	76.8
**SEC. TRANSPORT**						**SEC. TRANSPORT**	
N	%					N	%
9	10.7					2	2.3

*ALS* approved landing site, *HLS* Heliport landings site

Due to extra patient transfers, the mean transportation time increased with 25 min compared to a landing close to the hospital. No complications due to adverse events occurred during these extra patient transfers.

The average transfer time from landing at the referral hospital and take-off to the receiving hospital is 62 min (SD 14 min). This is 58 min (SD 10 min) at the receiving hospital.

## Conclusions

The construction of pre-explored and approved landing sites in the vicinity of ICU’s of hospitals allows safe transportation of ventilated COVID-19 IC-patients. In order to prevent delay secondary transport should be avoided. Landscape architects should be encouraged to consider the design of the hospital surroundings in such a way that an ALS fits in the natural landscape. Not many architectural adaptations are necessary nor are big financial investments necessary. Early consultation of HEMS-helicopter providers is advisable.

## Data Availability

Please contact author for data requests.
